# Dioscin ameliorates slow transit constipation in mice by up-regulation of the BMP2 secreted by muscularis macrophages 

**DOI:** 10.22038/IJBMS.2022.64683.14236

**Published:** 2022-09

**Authors:** BingBing Ren, SiQi Fu, Yong Liu, JianYu Kang, Bo Wang, ZhiWei Yao, Hao Wang, DaQing Sun

**Affiliations:** 1 Department of Pediatric Surgery, General Hospital, Tianjin Medical University, Tianjin, China; 2 Department of General Surgery, General Hospital, Tianjin Medical University, Tianjin, China; # These authors contributed eqully to this work

## Abstract

**Objective(s):**

The loss of enteric neurons has been shown to be a major cause of slow transit constipation (STC). Gut microbiota and muscularis macrophages (MMs) are associated with the enteric nervous system (ENS) development and gastrointestinal (GI) motility. This study aimed to investigate whether Dioscin (DIO) increased GI motility and inhibited neuron loss by modulating gut microbiota profile, improving inflammation in the ENS microenvironment.

**Materials and Methods:**

The STC model was established by loperamide. The alteration of the gut microbiota was analyzed by 16S rDNA sequencing. The longitudinal muscle and myenteric plexus (LMMP) from the colon were prepared for flow cytometry, immunofluorescence, western blot, and qRT-PCR.

**Results:**

DIO increased the stool number, stool water content and shortened whole gut transit time, helped to recover the gut microbial diversity and microbiota community structure, and increased the abundance of Muribaculaceae in STC mice. Compared with the STC group, the number of MMs and the level of the iNOS, IL-6, and TNFα genes were significantly decreased following DIO treatment. Moreover, DIO may increase the number of HuC/D^+^ neurons per ganglion by up-regulating the BMP2 secreted by MMs and activating the BMP2/p-Smad1/5/9 signaling pathway. Furthermore, the level of excitatory neurotransmitter AchE in colon tissues exhibited a substantial increase in the DIO group. However, the level of inhibitory neurotransmitter VIP was markedly decreased.

**Conclusion:**

Our results provide that DIO increases GI motility and inhibits neuron loss by modulating gut microbiota profile, improving inflammation in the ENS microenvironment and up-regulating the BMP2 secreted by MMs.

## Introduction

Functional constipation is a common chronic gastrointestinal symptom that is associated with multiple health problems such as colorectal neoplasms, ischemic stroke, and coronary heart disease (1, 2). With the change in dietary patterns and lifestyle, the incidence of constipation is increasing year by year, which has a negative impact on people’s quality of life (3). Slow transit constipation (STC) is a common subtype of functional constipation that is characterized by decreased phasic colonic motor activity and reduced frequency of defecation. The enteric nervous system (ENS) plays an important role in regulating gastrointestinal (GI) motility. ENS mainly consists of the submucosal (Meissner’s) and the myenteric (Auerbach’s) plexus. However, bowel relaxation and contraction are mainly controlled by the myenteric plexus. Numerous macrophages accumulate in the muscular layer of the gut wall which is known as muscularis macrophages (MMs). MMs are important for gastrointestinal neuromuscular function. The MMs regulate GI motility by secreting BMP2 which contributes to ENS development (4). In addition, MMs display an anti-inflammatory M2 phenotype. A shift in macrophage polarization from anti-inflammatory ‘M2’ to pro-inflammatory ‘M1’ state is associated with chronic low-grade inflammation in the ENS microenvironment and colon delayed transit (5). Recent evidence has shown that the number of MMs is elevated in colonic specimens from STC patients (6). In addition, intestinal neuronal apoptosis was significantly higher in STC patients than in controls (7). Our previous research found that nitrergic enteric neurons were significantly increased in STC patients compared with normal control (8). However, the current drugs for constipation are mostly osmotic or secretory laxatives including senna, bisacodyl, and prucalopride. Long-term use of laxatives results in ENS damage, QT prolongation, melanosis coli, etc. (9-11). Therefore, there is a need to find an effective therapy for constipation without side effects.

Due to the safety and laxative effect of natural products, various herbal medicines are considered, promising treatments for constipation. Dioscin (Dio), a kind of steroidal saponins, exists in some medicinal plants and has multiple medicinal efficacies. Dio has been proven to have anti-inflammatory, anti-oxidative stress, and anti-tumor activities (12-14). Moreover, Dio can target macrophages and decrease pro-inflammation cytokines IL-6, IL-1β, and TNF-α to ameliorate lung fibrosis (15). In addition, Dio can significantly ameliorate neuroinflammation and oxidative damage of neurons (14). The neuroinflammation and oxidative damage of neurons play an important role in the onset of constipation (5, 16). A study found that the Chinese yam extract containing Dio increases gastrointestinal motility and affects the conversion of some intestinal flora to helpful bacteria (17). In this study, we will further study whether DIO can increase GI motility and investigate the underlying mechanisms through which DIO ameliorates loperamide-induced constipation symptoms.

## Materials and Methods

***Animals and experimental design***

Thirty 8-week-old Kunming mice weighing 35±2g were purchased from BEIJING HFK BIOSCIENCE Co. Ltd. with License key of SCXK 2020-0004. All mice were provided with a standard commercial mouse diet and water as well as a 12/12 hr light-dark cycle at the Tianjin General Surgery Institute, China. All animal experimental operations for this study were approved by the Ethics Committee of Tianjin Medical University (No. IRB2021-DW-30) and were reported in accordance with the ARRIVE guidelines. 

After 5 days of adaptation, 30 mice were randomly divided into three groups (n=10 per group): normal control group (CON); slow transit constipation model group (STC); DIO group of constipation mice treated with Dioscin (Spring & Autumn Biological Engineering Co. Ltd, Nanjing, China). All groups except the CON group were given loperamide (Sigma St. Louis, MO, USA) by gavage at 10 mg/kg twice per day for 12 days. DIO group was treated with Dioscin at 60 mg/kg from day 6 to 12. The CON and STC group were treated with saline as vehicle by gavage. The food intake of each group was measured every day and the weight of the mice was measured every three days. 

***Measurement of feces parameters***

After day 12 of gavage administration, all mice were fasted for 24 h. On day 13, all mice were given 0.2 ml of India ink (0.4 mg/ml, Shanghai yuanye Bio-Technology Co., Ltd) to determine the time of first black stool defecation which was used to evaluate the whole gut transit time. The stool of the individual mouse was collected during the 6 hr after being given India ink. Then the stool number and wet weight were recorded. The fecal pellets were dried at 60 °C for 24 hr. The fecal water content was calculated according to the following formula:

fecal water content = (wet weight-dried weight)/ wet weight × 100

***Histopathological examination***

Mouse distal colonic tissues were fixed with 4% paraformaldehyde and embedded in paraffin. Then, colonic tissues were sectioned into 5 µm-thick sections, which were deparaffinized and stained with Hematoxylin and Eosin (H&E, Solarbio, Beijing, China). Six tissue segments per group were prepared and histological morphology was observed by light microscopy. 

***Interaction analysis of DIO with related proteins***

We analyzed the interaction data of the active ingredient and the putative target of DIO for the treatment of loperamide-induced constipation. Protein targets were retrieved from the TCMSP database (http://lsp.nwsuaf.edu.cn/tcmsp.php). The R package clusterProfiler was used to annotate GO terms associated with biological processes (BP).

***16S rDNA microbial profiling analysis***

We investigated the change in gut microbiota by using a 16S rDNA sequencing technique. Firstly, DNA from mouse fecal samples was extracted using the E.Z.N.A. ®Stool DNA Kit (D4015, Omega, Inc., USA), following the manufacturer’s instructions. The specific primers targeting the V3 -V4 regions were used to amplify the bacterial 16S rDNA. The PCR products were sequenced using the NovaSeq PE250 platform. Raw reads from sequencing were filtered to obtain high-quality clean tags. Then, chimeric and replicate sequences were excluded to obtain the feature table and feature sequence. α-diversity including Chao1, Goods coverage, observed species, Shannon, and Simpson were calculated by QIIME2. The principal coordinates analysis (PCoA) plots were used to evaluate β-diversity. 

***LMMP preparation***

The longitudinal muscle and myenteric plexus (LMMP) were prepared as described previously (18). Briefly, the colon was cut into about 3 cm pieces and attached to a silicone plate in ice-cold PBS. Beginning at one end of the segment on the serosal surface, the LMMP was separated from the other gut layers under the microscope (ZEISS, S7/OPMI Sensera) by using fine forceps. The LMMP was saved at −80°C for western blot, qRT-PCR, and fixed in 4% paraformaldehyde for immunofluorescence.

***Flow cytometry***

The numbers of MMs were analyzed by Flow cytometry*.* LMMP was digested using a protocol adapted from Joseph *et al.* (19). Dissociated cells were obtained as previously described (5), which were stained with specific antibodies. First, the cell suspension was blocked with FcBlock (anti-CD16/CD32; BioLegend) for 10 min at 4 °C and subsequently stained with the F4/80 FITC antibody (BioLegend, San Diego, CA, USA) for intracellular staining the cell suspension. Flow cytometry analysis was performed using a BD FACSCanto II (Becton Dickinson, NJ, USA). A minimum of 20,000 events were used for each measurement. In FlowJo, the forward scatter (FSC-A) and side scatter (SSC-A) parameters were used to ensure that all populations of interest are visible. In a second step, F4/80 expression was analyzed in all live cells. 

***Quantitative PCR analysis***

Total RNA was extracted from LMMP using TRIzol Reagent (BS259A, Biosharp) according to the manufacturer’s instructions. The RNA concentration and purity were determined using a Nanodrop One spectrophotometer (Thermo Fisher Scientific Inc.). Then cDNA was subsequently synthesized using Hifair® III 1st Strand cDNA Synthesis Kit (11139ES10, YEASEN) according to the manufacturer’s protocols. The same amount of RNA (1 μg) from the sample was added to 3 μl 5×g DNA Digester Mix and 11 μl RNase-free H_2_O for 2 min at 42 °C. Next, the cDNA was synthesized in 20 μl body fluid which included 15 μl of the reaction liquid from the previous step, 5 μl 4×Hifair® III Super Buffer plus for 5 min at 25 °C, for 15 min at 55 °C, and 5 min at 85 °C. Q-PCR was performed using a cDNA template and the Hieff® qPCR SYBR Green Master Mix Kit (1184ES03, YEASEN). The primer sequences are presented in Table 1. The genes were amplified by subjecting the samples to 2 min at 95 °C, 40 cycles of 10 sec at 95 °C, and 30 sec at 60 °C. The expression level of GAPDH mRNA was used as endogenous reference control. The relative expressions of target mRNA were normalized to the control group by determining the 2-ΔΔCT values.

***Western blotting***

 Total protein was extracted from LMMP in radio-immunoprecipitation (RIPA) buffer containing 1% protease inhibitor cocktail by tissue homogenizer. The protein concentration was estimated using the BCA Protein Assay Kit (Solarbio, Beijing, China). The concentration of LMMP lysates was diluted to 2 ug/μl. Then, 15 μl aliquots of each sample were resolved on 10% sodium dodecyl sulfate-polyacrylamide gels (SDS-PAGE) for 2.5 hr, and proteins were transferred to PVDF membranes at 250 mA for 80 min. The PVDF membranes were sealed with 5% non-fat dry milk for 1 hr, incubated with primary antibodies overnight at 4 °C, then incubated with horseradish peroxidase-conjugated goat anti-rabbit antibody (1:2000 #ab6721, Abcam) for 2 hr at room temperature. Primary antibodies targeting BMP2 (1:1000 #ab214821, Abcam), p-Smad1/5/9 (1:1000 #13820, Abcam), β-Actin (1:5000 #ab179467, Abcam) were used as the loading control.

***Immunofluorescence***

LMMP was fixed in 4% paraformaldehyde overnight at 4 °C and washed 3 times (5 min/per time) by PBS. Then, LMMP was incubated with 0.5% Triton at RT for 1 hr, before being blocked in PBS containing 5% normal goat serum for 1 hr. LMMP was incubated with primary antibody anti-HUC/D (Invitrogen; 1:200) which was a pan-neuronal marker, anti-neuronal nitric oxide synthase (nNOS) (Invitrogen;1:300), at 4 °C for 2 days. After washing with PBS, the LMMP was incubated with appropriate secondary antibodies at RT for 2 hr and overlaid with coverslips onto glass slides using mounting media with DAPI. Slides were imaged with a CCD camera on an OLYMPUS BX51 microscope using a 20X objective. Then, we randomly selected a minimum of 10 myenteric ganglia and counted the number of HuC/D^+^ and nNOS^+^ neurons per myenteric ganglia in each mouse.

***Gastrointestinal hormone assay***

After the mice were sacrificed, the colon tissue was collected and stored at −80 °C for subsequent experiments. An appropriate amount of colon tissue was disrupted by using a homogenate machine in corresponding volume of PBS and centrifuged to obtain the supernatant. The levels of acetylcholinesterase (AchE), substance P (SP), and vasoactive intestinal peptide (VIP) in the colon tissue were determined using corresponding ELISA kits (96T, Tianjin Anoric Bio-technology Co. Ltd) according to the manufacturer’s instructions. 

***Statistical analysis***

The statistical analysis and histograms were processed with GraphPad Prism 9. Quantitative data were expressed as the mean ± SD. The significant differences between groups were determined by one-way ANOVA followed by the least significant difference (LSD). *P*<0.005 was considered statistically significant. 

## Results

***Effects of DIO on mouse weight and feces parameters***

**Figure 1:**
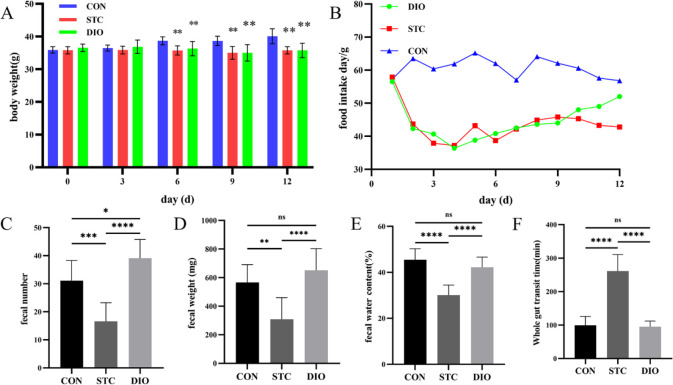
Effect of dioscin on feeding conditions, fecal excretion, and time to the first black stool in loperamide-induced STC mice. (A) Weight of the mice was measured every three days during the experiment. (B) Food intake of each group was measured every day during the experiment. (C) Number of feces, (D) Weight of feces, (E) Fecal water content, and (F) Whole gut transit time. *P<0.05 and **P<0.01

After day 6 of gavage loperamide, the bodyweights of the DIO group and STC group were significantly less than the CON group (Figure 1A). However, until the end of the treatment, the bodyweights did not differ significantly between the DIO and STC groups (Figure 1A). After day 3 of gavage loperamide, the food intake of DIO group and STC group was significantly decreased (Figure 1B). After treatment with DIO, the number and weight of feces were significantly increased (Figure 1C-D). In addition, the fecal water content was almost recovered in the DIO group compared with the CON group (Figure 1E). Time to the first black stool of the STC group was significantly longer than that of the CON and DIO group (Figure 1F). However, there was no statistical difference between DIO group and CON group. So, these results suggest that DIO can ameliorate loperamide-induced constipation symptoms in mice. 

***Effects of DIO on histological alterations***

**Figure 2:**
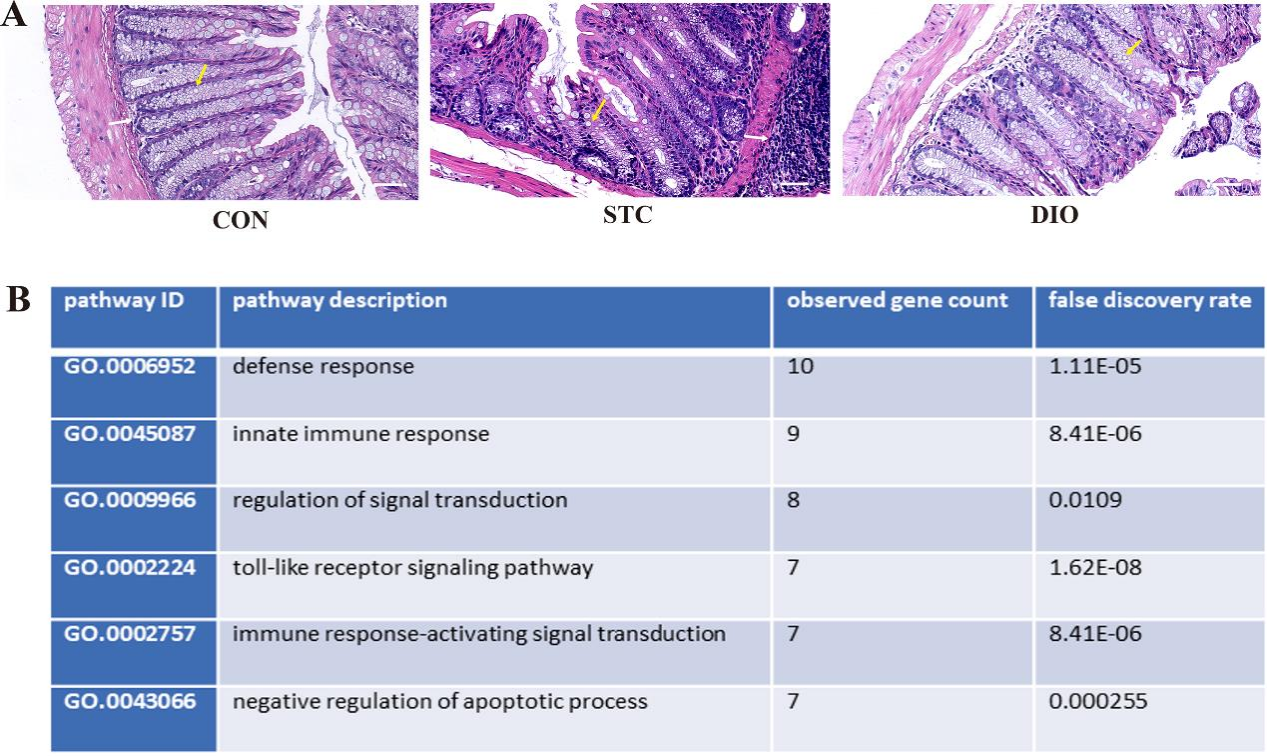
(A) HE staining was performed for estimating the effect of dioscin on histological profiles in the distal colon (magnification, 200×). (B) Gene Ontology (GO) analysis of the biological processes related to dioscin. White arrows indicate inflammation cell infiltration and yellow arrows indicate goblet cells. Scale bars, 50 μm

STC: Slow transit constipation; DIO: Dioscin; CON: Control

HE staining was used for estimating the effect of DIO on histological alterations in the distal colon. Figure 2A shows that the STC group exhibited a marked inflammation and significant reduction of goblet cells in the distal colon. However, DIO can improve inflammation and increase goblet cells. 

***DIO targets the innate and adaptive immune systems***

We investigated the underlying mechanisms through which DIO ameliorates loperamide-induced constipation symptoms in mice by using the TITCH database which is used to explore known and predicted interactions between chemicals and proteins. Then, Gene Ontology (GO) analysis was performed and we found that the inflammatory response may be involved in the biological effects of dioscin (Figure 2B).

***DIO altered microbiota structure of loperamide-induced STC mice***

**Figure 3:**
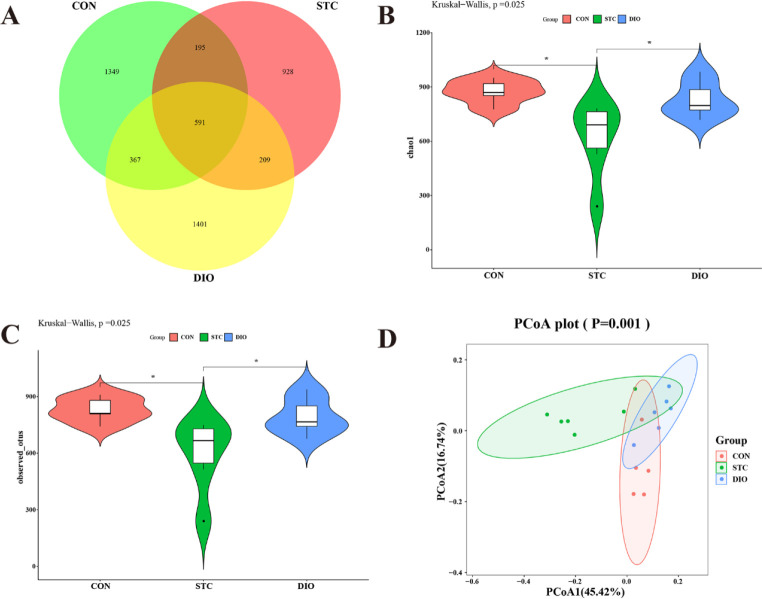
Modulating gut microbiota profile by dioscin in the feces of mice. (A) Venn diagram showing the OTU numbers identified in the three groups. (B-C) Chao 1 and observed species indexes reflect species diversity within the group. (D) Principal coordinates analysis (PCoA) plots were used to evaluate β-diversity. β-diversity reflects species diversity between the groups. *P<0.05

STC: Slow transit constipation; PCoA: Principal coordinates analysis; OUT: Operational taxonomic unit

To evaluate the alteration of microbiota structure of the loperamide-induced STC mice treatment with DIO, seventeen samples from the three groups were evaluated using 16S rDNA sequencing. A total of 4675 operational taxonomic units (OTUs) were identified among the three groups, including 2506 OTUs in the CON group, 1681 OTUs in the STC group, and 2263 OTUs in the DIO group (Figure 3A). Chao1 and Observed Species indexes of the CON and DIO groups were significantly higher than those of the STC group (*P*<0.05). In addition, there was no statistical difference between the CON and DIO groups (Figure 3B-C). These results suggested that the alpha diversity in the STC group was significantly lower. But the administration of DIO led to a significant increase in alpha diversity. The principal coordinates analysis (PCoA) plots were used to evaluate β-diversity. The weighted unifrac PCoA analysis showed that the microbiota community structure of the DIO group was more similar to the CON group, compared with the STC group (Figure 3D). 

**Figure 4:**
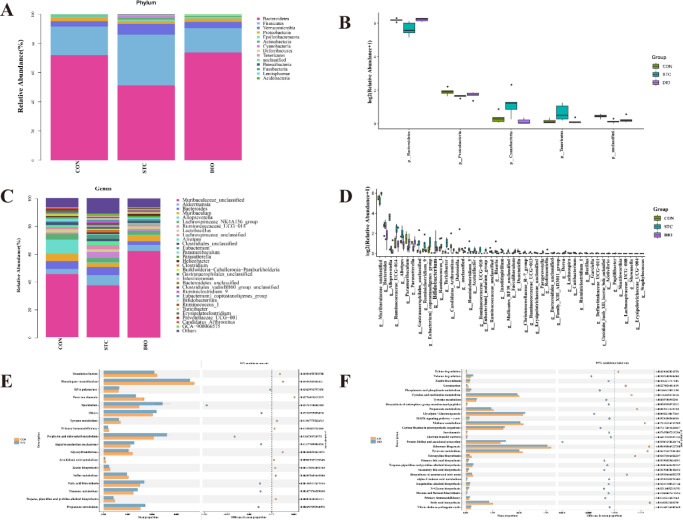
Alterations of gut microbiota at phylum and genus levels and gut bacterial function prediction analysis in the feces of mice. The relative abundances of gut microbiota in phylum level (A), and genus level (C). (B) The 5 taxa with the most obvious differences in phylum level between groups. (D) The 30 taxa with the most obvious differences in genus level between groups. (E and F) The gut bacterial function prediction analysis using PICRUST software. (E) Differentially metabolic pathways between the CON and STC groups, (F) Differentially metabolic pathways between the STC and DIO groups

STC: Slow transit constipation; DIO: Dioscin; CON: Control

We further investigated the alterations of gut microbiota at phylum and genus levels. At the phylum levels, we observed that Bacteroidetes, Firmicutes, Verrucomicrobia, and Proteobacteria were the dominant phylum (Figure 4A-B). Bacteroidetes accounted for the highest proportion which was significantly reduced in the STC group compared with the CON group. But administration of DIO led to a significant increase in the relative abundance of Bacteroidetes. Moreover, a significant increase in the abundance of Firmicutes and Cyanobacteria in the STC group was found, also a decrease in these phylums’ abundance in feces after treatment with DIO. At the genus level, Muribaculaceae was the most abundant genera. The relative abundance of Muribaculaceae in the STC group was lower than that of the CON group but was significantly increased by DIO treatment. In addition, a significant decrease in the abundance of Parasutterella and Paramuribaculum in the STC group was found, and an increase of these genera’s abundance in feces by DIO treatment. Compared with the CON group, the abundance of Ruminococcaceae_UCG-014 in the STC group was increased. The treatment with DIO can make the abundance of Ruminococcaceae_UCG-014 decrease (Figure 4C-D).

Then, PICRUST software was used to perform gut bacterial function prediction analysis. In addition, the differentially metabolic pathways were got by comparing them with the KEGG database. Figure 4E and Figure 4F respectively showed the differential metabolic pathways between the CON and STC groups and between the STC and DIO groups. We found six differentially metabolic pathways which existed at the same time in Figures 4E and 4F. The six differentially metabolic pathways were associated with tyrosine metabolism, primary immunodeficiency, zeatin biosynthesis, fatty acid biosynthesis, propanoate metabolism, and tropane, piperidine, and pyridine alkaloid biosynthesis.

***Effects of DIO on the muscularis macrophages and ENS microenvironment***

**Figure 5:**
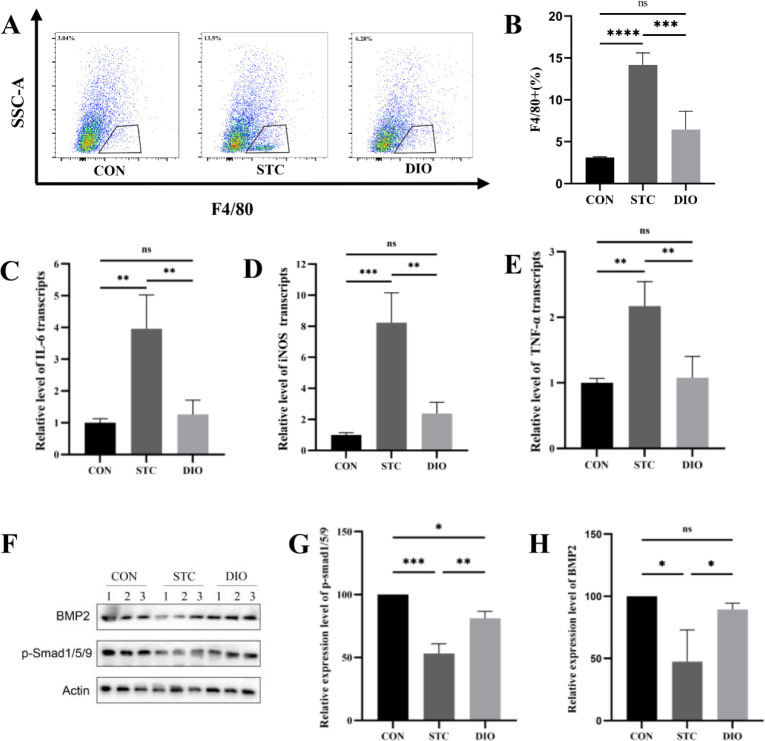
Effects of DIO on muscularis macrophages and ENS microenvironment. (A and B) Percent of MMs (F4/80+ cells) in all live cells. (C, D, E) mRNA expression levels of iNOS, IL-6, and TNFα in the muscularis layer of colon tissues. (F, G) protein expression levels of BMP2 and p-Smad1/5/9 in the muscularis layer of colon tissues. *P<0.05, **P<0.01, *** *P*<0.001, and **** *P*<0.0001

DIO: Dioscin; ENS: Enteric nervous system; MMs: Muscularis macrophages

Combining the results of GO analysis and gut bacterial function prediction analysis, we suggested that DIO may increase GI motility by improving inflammation. But, whether DIO improved inflammation in the ENS microenvironment was unclear. So, our study focused on the change of MMs. LMMP was digested as a single cell suspension to analyze the numbers of MMs by Flow cytometry. MMs, defined as F4/80+ cells, the numbers of which significantly increased in the STC group which comprised about 15% of total live cells. However, the number of MMs statistically reduced following DIO treatment (Figure 5A-B). Due to muscularis, macrophages preferentially express M2 markers (20). Next, we performed mRNA expression analysis of a variety of pro-inflammatory cytokines including iNOS, IL-6, and TNFα. These pro-inflammatory cytokines were statistically significantly increased overall in the ENS microenvironment in STC mice. Compared with the STC group, the levels of the iNOS, IL-6, and TNFα genes were significantly decreased following DIO treatment (Figure 5C-E). The results showed that DIO can improve inflammation in the ENS microenvironment.

***Effect of DIO on BMP2/p-Smad1/5/9 Pathway in LMMP Tissue***

MMs contribute to ENS development and regulate GI motility by secreted BMP2(4). So, the expression levels of BMP2 and p-Smad 1/5/9 were detected by western blot. BMP2 and p-Smad 1/5/9 protein expression levels were significantly lower in the STC group compared with CON. In addition, DIO groups elevated BMP2 and p-Smad 1/5/9 protein expression levels (Figure 5F-H).

***Effect of DIO on ENS***

**Figure 6:**
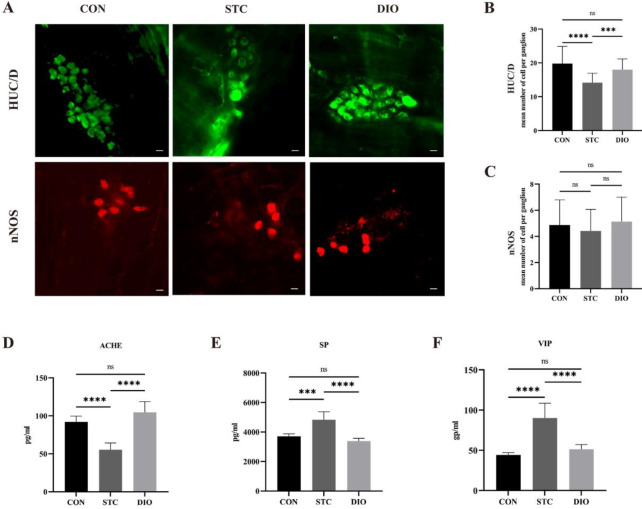
Effect of DIO on ENS. immunohistochemistry of LMMP was performed, (A, B, C) Number of HuC/D+ and nNOS+ neurons per ganglion. (D, E, F) Levels of neurotransmitters including AchE, SP VIP in the colon tissues of mice. *P<0.05, **P<0.01, *** *P*<0.001, and **** *P*<0.0001. Scale bars, 10 μm

DIO: Dioscin; ENS: Enteric nervous system; LMMP: Longitudinal muscle and myenteric plexus

In order to evaluate whether DIO reverses the changes of ENS, immunohistochemistry of LMMP was performed. Compared with the CON group, the number of HuC/D^+^ neurons per ganglion significantly decreased in the STC group. After DIO treatment, there was an increase in the number of HuC/D^+^ neurons per ganglion (Figure 6A-B). But, there was no statistical difference in the number of nNOS^+^ neurons per ganglion among the three groups (Figure 6A, C). 

***AchE, SP, and VIP levels in colon tissue***

The ACHE level of the STC group was significantly lower than that of the CON group (*P*<0.05). Compared with the STC group, the ACHE level markedly increased in the DIO group (*P*<0.005) (Figure 6 D). VIP and SP levels of the STC group were significantly higher than that of the CON group (*P*<0.005). Furthermore, compared with the STC group, VIP and SP levels exhibited a substantial decrease in the DIO group (*P*<0.05) (Figure 6E-F). In addition, the levels of ACHE, and SP VIP in the colon tissues of mice in the DIO groups were closest to those in the CON group.

**Table 1:** Sequences of primers used in this study for qPCR

**Gene Name**	**Sequence**
IL-6	Forward: 5’-CCGGAGAGGAGACTTCACAG-3’
	Reverse: 5’-TCCACGATTTCCCAGAGAAC-3’
TNFα	Forward:5’-CAGGCGGTGCCTATGTCTC-3’
	Reverse: 5’-CGATCACGAAGTTCAGTAG-3’
iNOS	Forward: 5’- ACCTTGTTCAGCTACGCCTT-3’
	Reverse: 5’- TCTTCAGAGTCTGCCCATTG-3’
GAPDH	Forward:5’- TGCACCACCAACTGCTTAG -3’
	Reverse: 5’- GATGCAGGGATGATGTTC -3’

STC: Slow transit constipation; DIO: Dioscin; CON: Control

## Discussion

The animal model of STC induced by loperamide has been widely used. Loperamide can inhibit colonic peristalsis and intestinal fluid secretion which decreases the number and water content of feces. In our study, the STC mice treated with DIO had increased number and water content of feces and shortened the whole gut transit time. So, DIO can ameliorate loperamide-induced constipation symptoms in mice. 

In recent years, many researchers have been concerned about the role of the gut microbiota in regulating gastrointestinal motility. Gut microbiota is essential for maintaining the normal development of ENS in adult mice (21). Lactobacillus rhamnosus GG reduced the whole gut transit time by increasing the expression of choline acetyltransferase (22). The loperamide-induced constipation model exhibited a significantly low microbial diversity and a high ratio of Firmicutes/Bacteroides, which was consistent with our results(23-25). In this study, DIO helped recover the gut microbial diversity and microbiota community structure in STC mice which may be an important mechanism of DIO alleviating constipation. Furthermore, Muribaculaceae has a negative correlation with the inflammation status of DSS-induced colitis (26). The relative abundance of Muribaculaceae in the STC group was lower which indicated that Muribaculaceae may be related to the inflammation status of STC mice. Therefore, when the abundance of Muribaculaceae increased, the inflammation was significantly improved in STC mice. We speculate that DIO increasing the abundance of Muribaculaceae may be a way to alleviate constipation. 

Next, we further explore the underlying mechanisms of DIO alleviating constipation by performing bioinformatics analysis using the TITCH database. We found that the inflammatory response may be an important biological effect related to DIO. Many previous studies had confirmed that the loperamide-induced constipation model exhibited a marked inflammation (27-29). These studies mainly focused on the macrophage cells distributed in the mucosa. However,GI macrophages are not only distributed within the mucosa but located in the muscularis. There are many studies that found that MMs play an important role in regulating GI motility. Due to MMs initially participating in inflammatory responses leading to postoperative ileus, depleting the MMs can alleviate postoperative ileus (30). To study the changes of muscular macrophages in STC mice, we firstly got LMMP from mice colons and analyzed the changes in the number of MMs by flow cytometry. Our findings showed that the MMs significantly increased in the STC group compared with the CON group. After being treated with DIO, the inflammation was significantly improved and the MMs significantly decreased compared with the STC group in this study. 

Gut macrophages play a strategic role in intestinal homeostasis. MMs preferentially expressed tissue-protective and wound healing genes or M2 (20). Furthermore, it has been shown that the number of MMs was positively related to intestinal motility (31). Due to investigating alterations in the phenotype of MMs, the expression levels of ‘M1’ markers including iNOS, IL-6, and TNFα were measured in the LMMP using qRT-PCR. We found that the expression levels of iNOS, IL-6, and TNFα were significantly increased in the STC group compared with the CON group. Moreover, after being treated with DIO, the expression levels of these genes were significantly decreased compared with the STC group. In this study, we first indicated that MMs preferentially expressed pro-inflammatory or M1 and affects the inflammatory milieu of the ENS microenvironment in STC mice. So, DIO can improve the ENS microenvironment and alter the phenotype of MMs. 

BMP2, a member of the transforming growth factor-β superfamily, has a beneficial effect on the differentiation and survival of enteric neurons (32, 33). The colonic slow transit was a major complication of the streptozotocin-induced diabetic model (34). In clinical settings, 60% of patients with diabetes in the US suffer from chronic constipation (35). Furthermore, decreased BMP2 expression and enteric neuronal loss were found in the intestine of diabetic rats (36). It was suggested that BMP2 can regulate GI motility. Due to secretion of BMP2 by MMs, we investigated the expression of BMP2. Our results demonstrated that the expression level of BMP2 significantly decreased in the STC group and the treatment of DIO increased the BMP2 protein level which is similar to the CON group. Thus, p-Smad1/5/9 was detected by western blot, which showed a marked decrease in the STC group as compared with the CON group. Furthermore, the treatment of DIO increased the p-Smad1/5/9 protein levels which are similar to the CON group. These results indicated that BMP2 signaling is activated in the DIO group. 

Neuronal loss within the ENS has been implicated in slow transit constipation (7, 37). The myenteric plexus plays a key role in coordinating bowel relaxation and contraction. However, the submucosal plexus is mainly involved in the regulation of epithelial secretion and blood flow. In this study, we mainly focused on the myenteric plexus and firstly investigated the changes of ENS in STC mice by immunofluorescence of LMMP. Consistent with other studies, our results showed that the number of HuC/D^+^ neurons statistically decrease in the STC mice (29). The HuC/D is a pan-neuronal marker. So, the number of HuC/D^+^ neurons represents the total number of neurons. After DIO treatment, a significant increase in the number of HuC/D^+^ neurons was observed compared with STC mice. Interestingly, there was no statistical difference in the number of nNOS+ neurons per ganglion among the three groups. But, the proportion of nNOS^+^ neurons in the STC mice was higher than that in the DIO group. The nNOS^+^ neurons represent nitrergic enteric neurons which release nitric oxide (NO) and induce colonic smooth muscle relaxation. Then, we investigated the changes in the neurotransmitters including SP, VIP, and AChE. SP is an excitatory neurotransmitter that not only stimulates gut movement but reduces inflammation and prevents intestinal damage in DSS-induced colitis (38). AChE is a kind of excitatory neurotransmitter that stimulates gut movement by binding to AChE receptors. VIP is a kind of inhibitory neurotransmitter that can relax colonic smooth muscle (39). Our results showed that the AChE levels in the colon of the STC group were statistically lower than those of the CON group, whereas the VIP and SP levels were higher than those of the CON group. After DIO treatment, the levels of these neurotransmitters were similar to the CON group.

## Conclusion

This study proved that DIO administration increased the number of fecal pellets, fecal water content, and shortened whole gut transit time, helping recover the gut microbial diversity and microbiota community structure, and increase the abundance of Muribaculaceae in STC mice. After being treated with DIO, the inflammation was significantly improved and MMs significantly decreased compared with the STC group. Then, we found that DIO may activate BMP2 signaling which recovers HuC/D^+^ neurons, increases the colon tissue levels of AchE, and decreases the colon tissue levels of VIP and SP. We postulate that DIO helped recover the gut microbial diversity and microbiota community structure, improve the ENS microenvironment, activate BMP signaling, remodel the myenteric plexus, and regulate the level of gastrointestinal hormones, thereby alleviating constipation.

## Authors’ Contributions

BBR and YL Conceived and designed the study. BBR and YL Performed the experiments. BBR and SQF Wrote the original draft. YL and SQF Analyzed the data and modified the manuscript. JYK and BW Helped build an animal model. JYK and ZWY Helped collect animal specimens. HW and DQS Helped modify the manuscript and provided financial support. 

## Availability of Data and Materials

The analyzed datasets generated during the present study are available from the corresponding author upon reasonable request.

## Ethics Approval and Consent to Participate

All protocols involving animals were approved by the Animal Care and Use Committee of Tianjin Medical University General Hospital (No. IRB2021-DW-30).

## Conflicts of Interest

All authors declare no conflicts of interest for this paper.
